# Bread-Making in Country Districts

**Published:** 1906-09-22

**Authors:** 


### BREAD-MAKING IN COUNTRY DISTRICTS.

In connection with the recent Bakers' Exhibition, concerning which so much praise was lavished on the huge firms and great companies exhibiting in the various departments, it is strange that in the several competitions how few prizes fell to the representatives of any great firms. In the competition for the manufacture of white bread open to the world the gold medal presented by the Directors of the International Bakers' Exhibition, held last week at the Islington Agricultural Hall, fell to a young man engaged in the baking industry at Delabole, a village in one of the least known districts in North Cornwall. The interest is enhanced by the fact that the prize awarded annually by the Hovis Bread Company for the manufacture of brown bread made by the specially prepared flour of that company, would also have been gained by that competitor but for the fact that he was debarred on the ground that he had won the championship in their competitions last year. He has gained this position for three successive years. Mr. Clements, of Mrs. Radcliffe and Son, Delabole, Cornwall, has to be congratulated on this effort. It is pleasant to consider, as guardians and exponents of the public health and interests that such success can only be due to the constant practice of baking for his customers bread that can stand the severe criticism of exhibition judges. After all country life in Delabole has its advantages in the best of milk, the richest of cream, the finest of butter, and championship bread.

The final competitions in bread have placed Mr. Bolton, of Clapham Park Road, in the proud position of winner of the sixty-guinea challenge cup offered by the National Association of Master Bakers and Confectioners, while he has also secured the county challenge gold medal. Moreover, he secures £200 offered by Messrs. Vernon, if the premier award was made with their Millennium flour, thus adding another to the long list of such triumphs that this firm has obtained. The winners of the horse and van, valued at £100, offered as the final prize in the comprehensive scheme of Hovis competitions, are Messrs. Child Brothers, of Croydon. That both these coveted distinctions should have been won within the area of Greater London was subsequently regarded by the experts as some answer to those critics who are wont to find fault with the standard of baking in the Metropolis.

